# Chlorhexidine–Silver Nanoparticle Conjugation Leading to Antimicrobial Synergism but Enhanced Cytotoxicity

**DOI:** 10.3390/pharmaceutics15092298

**Published:** 2023-09-09

**Authors:** Nadezhda Ivanova, Neli Ermenlieva, Lora Simeonova, Iliyan Kolev, Iliya Slavov, Daniela Karashanova, Velichka Andonova

**Affiliations:** 1 Department of Pharmaceutical Technologies, Faculty of Pharmacy, Medical University of Varna, 9000 Varna, Bulgaria; velichka.andonova@mu-varna.bg; 2 Department of Microbiology and Virology, Faculty of Medicine, Medical University of Varna, 9000 Varna, Bulgaria; neli.ermenlieva@mu-varna.bg; 3 Department of Virology, The Stephan Angeloff Institute of Microbiology, Bulgarian Academy of Sciences, 26 G. Bonchev Str., 1113 Sofia, Bulgaria; lora_simeonova@microbio.bas.bg; 4 Department of Pharmaceutical Chemistry, Faculty of Pharmacy, Medical University of Varna, 9000 Varna, Bulgaria; ilian.kolev@mu-varna.bg; 5 Department of Biology, Faculty of Pharmacy, Medical University of Varna, 9000 Varna, Bulgaria; ilia.slavov@mu-varna.bg; 6 Institute of Optical Materials and Technologies “Acad. Jordan Malinowski”, Bulgarian Academy of Sciences, Acad. Georgi Bonchev Str., bl. 109, 1113 Sofia, Bulgaria; dkarashanova@yahoo.com

**Keywords:** silver nanoparticles, chlorhexidine, conjunction, complexation, surface functionalization, nano-scaled drug delivery, antimicrobial synergism, antiviral activity, *Camellia sinensis* catechins, green synthesis

## Abstract

This study explored the potential synergism within chlorhexidine–silver nanoparticle conjugates against Influenza type A, *Staphylococcus aureus*, *Escherichia coli*, and *Candida albicans*. Silver nanoparticles (SN) were obtained by the reduction of silver ions with green tea total phenolic extract and conjugated with chlorhexidine (Cx). The particles were characterized by UV-Vis and FTIR spectroscopies, dynamic light scattering, X-ray diffraction, and transmission electron microscopy. A stable negatively charged nano-silver colloid (ζ = −50.01) was obtained with an average hydrodynamic diameter of 92.34 nm. In the presence of chlorhexidine, the spectral data and the shift of the zeta potential to positive values (ζ = +44.59) revealed the successful sorption of the drug onto the silver surface. The conjugates (SN-Cx) demonstrated potentiation in their effects against *S. aureus* and *C. albicans* and synergism against *E. coli* with minimal inhibitory concentrations of SN at 5.5 µg/mL + Cx 8.8 µg/mL. The SN showed excellent virucidal properties, increasing with time, and demonstrated low toxicity. However, the coupling of the cationic chlorhexidine with nano-silver did not reduce its intrinsic cytotoxicity on various cell lines (MDCK, BJ, and A549). The newly synthesized antimicrobial agent exhibited an extended and promising therapeutic spectrum and needs to be further evaluated regarding the designated route of administration in three-dimensional cell models (e.g., nasal, bronchial, dermal, ocular, etc.).

## 1. Introduction

In the last few years, several reports have become available concerning the mutual or parallel utilization of silver nanoparticles (SN) and chlorhexidine (Cx) as a treatment/prophylactic strategy for specific infectious diseases. Both agents are widely studied and described in the literature as possessing distinct antimicrobial properties, which made a lot of scientists curious about a possible synergism, additive effects, or potentiation of their activities. Some interesting findings were reported by Zhou et al., who prepared a composite of poly(L-lactide) microcapsules of chlorhexidine with superficially adsorbed silver nanoparticles and found better antibacterial properties against *E. coli* and *S. aureus* with less usage of silver, compared to silver nanoparticles carrying non-drug loaded microcapsules [1]. Elsewhere, Myronov et al. reported that adding silver nanoparticles to chlorhexidine treatment improved infected wound healing through the acceleration of bacteria elimination (*E. coli*, *P. aeruginosa*, and *S. aureus*) and M2 macrophage polarization [2]. Pernakov et al. established the accelerated wound healing effect of silver nano-architectures and chlorhexidine when combined, compared with single treatments with both agents, but did not observe any synergism in the antibacterial activity against *E. coli* and *P. aeruginosa* [3]. Other authors reported synergism or the potentiation of SN and Cx against *Candida* spp. [4,5,6], *Enterococcus faecalis* [5,7], *Klebsiella pneumonia* [5], *Pseudomonas aeruginosa* [6,8], *Staphylococcus epidermidis* [6,8], and *Streptococcus mutans* [6,9]. To the best of our knowledge, yet to be cursorily addressed are the supramolecular interactions that may occur upon the addition of chlorhexidine to a nano-silver colloid, along with defining the nature of the resultant effects. It would be speculative to seek any controversy or agreement in all the findings above since the surface functionality of nano-silver is indeed the crucial factor for accessing their potential physicochemical and therapeutic properties. It depends highly on the preparation technique and the reducer that is used [10,11,12,13].

Today, due to their pronounced antibacterial, antifungal, antiviral, and anticancer properties, SN are considered highly potent multifunctional units with a broad application range (e.g., in nanomedicine, dentistry, ecology, the textile industry, cosmetics, etc.) [14,15,16,17]. Their antimicrobial activity is primarily owing to the silver ions (Ag^+^) released from the critically increased silver surface and subsequent oxidation or bonding to essential for the microorganisms’ vitality, adhesion, and replication molecular factors [18,19,20]. Ever since “green” synthetic methods were introduced, it has become a trend and a method of choice to obtain nano-silver colloids by reducing silver ions in dilute solutions of soluble silver salts with non-toxic reducers of natural origin—plant extracts, bacteria, yeast, and fungi [11,21]. Being a rich source of primary and secondary metabolic products, most of them serve not only as reducers but also as capping (stabilizing) agents [11,22]. However, for the same reason, the various biological methods lead to highly differentiating functionality (shape, size, charge, redox potential, and superficial organic load or “cap”) of the silver nanoparticles, affecting the efficiency of Ag^+^ release. Thus, depending on the reduction process and the surface properties, SN possess varying antimicrobial strength, toxicity, and, last but not least, the ability to conjugate with other substrates [11,23].

Chlorhexidine (Cx) is a broad-spectrum topical antiseptic with a biguanide structure and the behavior of a cationic surfactant in neutral and alkaline media. The antifungal properties of the compound are slightly less pronounced than its antibacterial properties [24,25]. Cx was considered a suitable substrate for conjugation to SN for several reasons: (1) when ionized, the molecule produces quaternary ammonium functional groups, which can easily contribute to the electrostatic adsorption of Cx on a negatively charged substrate [26,27] (such as green-synthesized nano-silver in most cases [28,29]); (2) the presence of multiple basic N atoms sets a prerequisite for coordination with the silver nuclei [30], whereas the acidic NH are expected to engage in hydrogen bonding with the available phenolic groups in the organic cap [31,32,33,34]. It is as yet unclear how these potential interactions may influence the therapeutic activity of SN and chlorhexidine itself, and whether they allow/determine potentiation or even synergistic action.

Our pilot study on “green” SN synthesis with green tea extracts confirmed the better stability and purity of nano-silver colloids when catechin-rich phenolic extract was used for the reduction of silver ions rather than using total aqueous green tea extract [35]. Furthermore, we obtained a clue regarding the synthesis’ optimal parameters and the colloidal stability. In the present study, we attempted to explore the production variables in more detail and establish the physicochemical nature of the resultant nano-silver colloids with a view to their conjunction with chlorhexidine. We hypothesized that in a conjugated form, Cx and SN may present synergistic activity against common pathogens, such as the Influenza virus, *Staphylococcus aureus*, *Escherichia coli*, and *Candida albicans*.

## 2. Materials and Methods

### 2.1. Materials

#### 2.1.1. Chemicals and Reagents

Sencha green tea was supplied by a local drugstore. Silver nitrate at >99.9% and sodium hydroxide pellets at >98% were purchased from Thermo Fisher Scientific, Oxford, UK. Chlorhexidine diacetate salt hydrate at ≥ 98%, Mw 625.55 g/mol, was supplied from Sigma Aldrich, Burlington, MA, USA; all organic solvents were purchased from Sigma-Aldrich (USA) and were of analytical grade.

#### 2.1.2. Bacteria and Fungi

The *Escherichia coli* ATCC 25922*, Staphylococcus aureus* ATCC 25923, and *Candida albicans* ATCC 10,231 reference strains (MicroSwab) were obtained from Ridacom, Sofia, Bulgaria. Brain Heart Infusion broth and blood–agar (HiMedia) were also purchased from Ridacom, Bulgaria.

#### 2.1.3. Virus

Allantoic fluid and the cell-derived seasonal Influenza virus laboratory strains A/Aichi/2/68 (H3N2) (D. I. Ivanovsky Institute, Moscow, Russia) and A/Panama/07/99 (H3N2) (National Center for Infectious and Parasitic Diseases—NCIPD, Sofia, Bulgaria) were used at working doses of 100 CCID_50_/mL (T = 10–5.5 lg CCID_50_/mL).

#### 2.1.4. Cells

Madine–Darby canine kidney (MDCK) (ATCC-CCL-34^™^) and human lung carcinoma A549 (ATCC-CCL-185™) epithelial cell lines were kindly provided by Dr. Cyril Barbesange of the National Reference Center for Influenza, Sciensano, Belgium, as well as human diploid fibroblast cells BJ (ATCC-CRL-2522^™^), kindly provided by Dr. Kameliya Vinketova of the Institute of Biology and Immunology of Reproduction “Acad. Kiril Bratanov” in the Bulgarian Academy of Sciences (IBIR-BAS), were used. Cells were seeded at a density of 2.5 × 10^5^/mL at 37 °C in a 5% CO_2_ incubator, the Thermo Forma 310 (Thermo Fisher Scientific, Waltham, MA, USA), as a monolayer culture in 96-well plates (Corning^®^ Costar^®^, Corning, NY, USA) in growth medium DMEM (Gibco, Waltham, MA USA) or RPMI (Sigma, Livonia, MI, USA) containing 5 and 10% fetal bovine serum (FBS) (Gibco), 3.7 mg/mL sodium bicarbonate, 10 µM HEPES buffer (AppliChem GmbH, Darmstadt, Germany), 100 U/mL penicillin, 100 µg/mL streptomycin, and 25 µg/mL amphotericin B for 24 h until monolayer confluence was reached.

### 2.2. Methods

#### 2.2.1. Green Tea Total Polyphenols Extraction

A catechins-rich total phenolic fraction from Sencha green tea was isolated, as reported previously [35]. For the purposes of the current study, a methodology described by Krishnaswamy was applied with slight modification [36]. Sencha green tea leaves were extracted with methanol, and the obtained filtrate was evaporated in a water bath. Then, 10 mL of water and sodium chloride at 10% were added to the dried extract. The mixture was placed in a separating funnel with 10 mL of ethyl acetate, then the combined ethyl acetate extracts were washed twice with water. After evaporation of the organic solvent, a mixture of catechins and other phenolic compounds was obtained.

#### 2.2.2. Synthesis of Silver Nanoparticles (SN)

Silver ions were reduced with green tea phenolic fraction, following the protocol established in our pilot study [35]. Briefly, silver nitrate (AgNO_3_) solutions (1 mM and 2 mM) were obtained by dissolving the salt into distilled water under manual stirring at room temperature and via proper dilution afterward. The reductant, subsequently referred to as C8, was prepared in the form of a green tea polyphenol aqueous solution with varying concentrations of 0.6–6.0 mg/mL and was then alkalized to a pH of 8.0 with the aid of sodium hydroxide 10% solution (10–40 µL). The reaction vials were filled with 3.0 mL of silver nitrate solution, to which 0.5 mL of reductant was added. The mixtures were sealed and briefly shaken to homogenize the contents. The vials were left to rest under dark conditions and at ambient temperature overnight.

The silver nanosuspensions were purified from low-molecular-weight unreacted phenolic compounds and ions through a dialysis procedure, using Spectra/Por^®^2 dialysis membrane MWCO 12–14 kDa (Spectrum Laboratories, Inc., Rancho Dominguez, CA, USA). The membranes were prepared as recommended by the manufacturer. The sample was transferred to a dialysis bag at a volume of 10.5 mL (the contents of 3 reaction vials), immersed into 1000 mL dialysate (distilled water), and left under continuous stirring for 24 h. The process was conducted at room temperature and the sample was protected from the light environment. The resultant purified colloidal dispersion was evacuated from the dialysis bag with the help of a sterile syringe and a needle.

To preselect the most promising formulations, the samples were observed both visually, for colloidal stability (color, transparency, opalescence, turbidity, etc.), and spectrally to confirm silver nanoparticle formation and their relative concentrations for 7 days.

#### 2.2.3. Determination of SN Concentration

The concentration of SN (mg/mL) was determined after the natural evaporation to dryness of 1.0 mL colloidal solution in an 8.0 cm in diameter open glass Petri dish. The dried sample was left in a desiccator for at least 1 h afterward. The change in mass of the glass Petri dish was recorded using an analytical balance. The experiment was performed in triplicate in a previously tempered room, with tempered samples at 25 °C.

#### 2.2.4. Conjugation of SN and Chlorhexidine

An optimized formulation of silver nanoparticles (SN) was subjected to conjugation with chlorhexidine (Cx). For this purpose, an aqueous solution of chlorhexidine acetate (Mw 625.55 g/mol) at 3.75 mg/mL was prepared on a magnet stirrer (IKA^®^ C-MAG HS 4, Staufen, Germany) and added in varying volumes (0.5, 1.0, 1.5 mL) to a single reaction vial containing 3.5 mL of purified nano-silver suspension. The mixtures were sealed, briefly shaken to homogenize, and left to rest under dark conditions and ambient temperature. Whenever, during the tests, it was necessary to make a comparison with the silver nanoparticles before conjunction, equal volumes of distilled water (0.5, 1.0, or 1.5 mL) were added to the latter so that the nano-silver concentration in both conjugated and non-conjugated samples would be the same. 

#### 2.2.5. UV-Vis Spectroscopy

UV-Vis scans of the samples were carried out with a T60 UV-Vis spectrophotometer (PG Instruments, Lutterworth, UK) in the spectral range from 220 to 800 nm; the spectral analysis was performed on the second day after preparation (i.e., after dialysis completion), and on the seventh day after preparation. Appropriate dilution (1:10 during SN optimization and 1:20 after conjunction with Cx) was carried out on all samples in advance. The total green tea phenolic extract and chlorhexidine acetate were also scanned for reference using the same spectral interval. UVWin 6.0 software was used for data curation and processing after taking the measurements in triplicate.

#### 2.2.6. Dynamic Light Scattering/Laser Doppler Electrophoresis

The techniques of multi-angle dynamic light scattering (MADLS) and laser Doppler electrophoresis (LDE) were applied for the colloidal characterization of preselected SN samples and SN-Cx conjugated samples according to average size (hydrodynamic diameter—d_H_), size distribution, polydispersity, and zeta potential, respectively. Both studies were performed on a Zetasizer Ultra Red (λ = 632.8 nm) (Malvern Panalytical Ltd., Malvern, UK) at 25 °C. Data was processed and obtained with the aid of the ZS XPLORER 3.2.0.84 software. The samples were scanned on the second day after preparation and without any preliminary filtration.

#### 2.2.7. Transmission Electron Microscopy (TEM)

TEM images were taken with a JEOL JEM 2100 (JEOL Ltd., Tokyo, Japan) transmission electron microscope at an accelerating voltage of 200 kV.

#### 2.2.8. FT-IR Study

The IR spectra were recorded on a Bruker Tensor II FTIR spectrometer in the 3750–400 cm^−1^ spectral region, accumulated via 32 scans at a resolution of 4 cm^−1^. A KBr tableting technique was used. The SN and SN-Cx samples were freeze-dried for the purpose without adding any stabilizers, e.g., cryoprotectants, lyoprotectants, etc.

#### 2.2.9. X-Ray Diffraction (XRD)

XRD analysis was performed on SN and SN-Cx freeze-dried samples in a Panalytical Empyrean diffractometer (Malvern Panalytical Ltd., Malvern, UK). Chlorhexidine acetate crystalline powder was scanned for reference. The diffractograms were obtained by using a Cu-Kα source (λ = 1.5406 Å). The measurements were performed at a 1 s/step scanning rate, with a step size of 0.013°.

#### 2.2.10. Antibacterial and Antifungal Activity

The antibacterial and antifungal activity of SN, Cx, and SN-Cx samples were tested against the *Escherichia coli* ATCC 25922, *Staphylococcus aureus* ATCC 25923, and *Candida albicans* ATCC 10,231 reference strains. The stock concentrations of the active agents were set at 0.7 mg/mL for SN samples, 1.13 mg/mL for Cx samples, and 0.7 mg/mL + 1.13 mg/mL for SN-Cx samples, respectively. A serial 2-fold dilution method was applied to eight test tubes in a row containing 1.0 mL Brain Heart Infusion (BHI) broth. The resultant test concentrations were in the range of a 1:2 to 1:256 dilution of the stock solutions. Next, 0.1 mL of microbial suspension standardized to 0.5 McFarland turbidity equivalent was added to the vials. Samples of 1.0 mL of BHI broth contaminated with 0.1 mL microbial suspension of each strain were used as a positive control, whereas negative controls were set by mixing 1.0 mL of BHI broth with each active agent. All tests were repeated in triplicate. The test vials containing *S. aureus* and *E. coli* were incubated for 24 h at 37 °C and those with *C. albicans* were incubated for 48 h at 35 °C. The minimal inhibitory concentration (MIC) of each substrate was determined thereafter as the lowest active concentration in which no visible turbidity was observed. The fractional inhibitory concentrations (FIC) of Cx and SN were calculated as:

FIC_Cx_ = MIC (Cx in the presence of SN)/MIC (Cx alone)

FIC_SN_ = MIC (SN in the presence of Cx)/MIC (SN alone)

The following values were taken into consideration in the interpretation of the results: an FIC index of <0.5 indicates synergism, while >0.5–1 shows additive effects, >1 to <2 shows indifference, and ≥2 is considered to be antagonism [37].

Minimal bactericidal/fungicidal concentrations (MBC/MFC) were determined by transferring all test suspensions with no visible turbidity in a single bacterial-loop volume onto blood agar. The specimens thus obtained were incubated again under the same conditions described above. The lowest concentration at which bacterial/fungal growth appeared to be inhibited 99.9% was reported as MBC/MFC.

#### 2.2.11. Cytotoxicity (CC_50_) and Antiviral Effect (IC_50_ and SI) Evaluation via a Neutral Red (NR) Uptake Assay

After infection of the cell monolayer and treatment with each of the active agents in a respective dilution of the stock solutions (starting from 1:10 and following a two-fold step), the 96-well plates (including the virus and toxicity controls) were incubated in a thermostat at 37 °C and 5% CO_2_ for 72 h. The treatment effect was first assessed visually under a microscope and then assessed by staining with NR dye [38]. The light absorbance of the samples was read at 540 nm on a microplate reader (Biotek Organon, West Chester, PA, USA).

The values that were obtained were used to calculate the percentage of cell protection by applying the formula:

% (CPE) inhibition = [OD_test sample_ − OD_virus control_]/[OD_toxicity control_ − OD_virus control_] × 100,

where CPE stands for cytopathic effect and OD stands for optical density.

Toxicity was calculated as a percentage of untreated cell controls. The values obtained were used to calculate CC_50_, IC_50_, and SI. The concentration of the substance that causes 50% damage to cells after treatment is referred to as the 50% cytotoxic concentration or CC_50_. The 50% inhibitory concentration (IC_50_) is defined as the concentration of the substance that reduces viral replication by 50% compared to the untreated viral control. The selectivity index (SI) is represented by the CC_50_/IC_50_ ratio.

#### 2.2.12. Determination of the Effect on Extracellular Virions—Virucidal Effect

Equivalent volumes of 100 CCID_50_/mL virus suspension and the test samples (SN, Cx, or SN-Cx) were contacted in a 1:1 ratio and incubated at room temperature for different time intervals (5, 15, 30, and 60 min). The active agents were applied at their maximum tolerated concentration (MTC) or in two-fold and, correspondingly, ten-fold dilutions, as follows:

- SN 35 μg/mL (MTC) or 350 μg/mL (2-fold dilution of stock solution);
- Cx 2 μg/mL (MTC) or 113 μg/mL (10-fold dilution of stock solution);
- SN-Cx 1.2/2 μg/mL (MTC) or 70/113 μg/mL (10-fold dilution of stock solution).

The samples were then titrated using the end-point dilution method in a 24-h cell monolayer, starting either directly from the contact sample (when applying MTC) or after an initial ten-fold dilution of the contact sample (incubated with 2-fold- or 10-fold-diluted stock solution), with the content of the residual infectious virus being assessed at 72 h post-infection by visual reading of the cytopathic effect under an inverted microscope and via an NR uptake assay. Differences in CPE, denoted as Δlg, were recorded between the treated groups and the control virus suspension, incubated for the same time intervals in the absence of the active agents.

#### 2.2.13. Statistical Data Processing

The experiments were performed in two and three replicates to ensure statistical reliability of the results. Data was processed using the software Gen5^®^ 2018, Microsoft Excel^®^ 2016, Origin 8.5^®^ and GraphPad Prism 6.0^®^.

## 3. Results and Discussion

### 3.1. Silver Nanoparticle Synthesis, Optimization, and Conjunction

**Figure 1:**
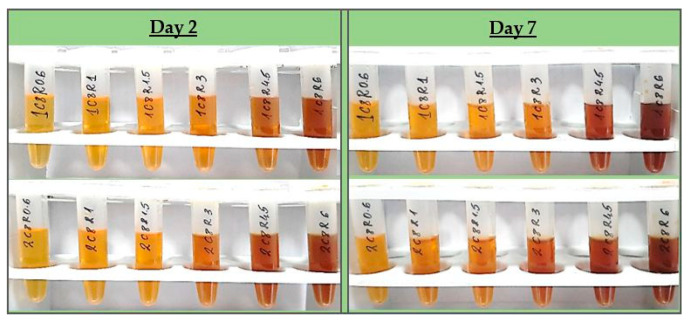
Visual appearance of SN samples on day 2 after preparation (immediately after dialysis) and on day 7 after preparation.

All test formulations resulted in the formation of nano-silver, recognized by the immediate change in color to the yellow-reddish-brown scale and the surface plasmon resonance (SPR) peak, manifesting in the range of 413–430 nm in all UV-Vis spectra. According to the spectral data (particularly, the absorption at the wavelength of the SPR extremum), the darkness of the color was found to correspond well with the concentration of the colloids. The highest nanosilver concentration with no visible signs of turbidity and/or sedimentation was established with sample 2C8R4.5, obtained in 2 mM of silver nitrate solution with green tea polyphenols (C8) at 4.5 mg/mL. Table 1 summarizes the information for all test compositions and the obtained UV-Vis spectral data; in Figure 1, the visual appearance of the samples over 7 days can be followed.

The colloidal stability of samples 2C8R [1.5–4.5] was confirmed via zeta potential analysis. With the zeta potential of sample 2C8R4.5 being the highest among all the samples according to absolute value [−50.01 mV], the initial observation was confirmed for it to be the composition of choice for further analyses and conjunction with Cx. Through evaporation to dryness, the nano-silver content (including the organic load) in the sample in question was established to be 1.005 ± 0.012 mg/mL.

As expected, upon the successful sorption of chlorhexidine diacetate (Cx) onto the silver nanoparticles’ surface, the colloids acquired a positive zeta potential; favorably for the purposes of this study, the latter was found to reach maximal absolute values in samples obtained with the highest investigated drug load, i.e., 1.5 mL of Cx 3.75 mg/mL solution. At such a dilution of the colloid, the nano-silver concentration drops to 0.7 mg/mL. Table 2 summarizes the data from the zeta potential measurements.

**Figure 2:**
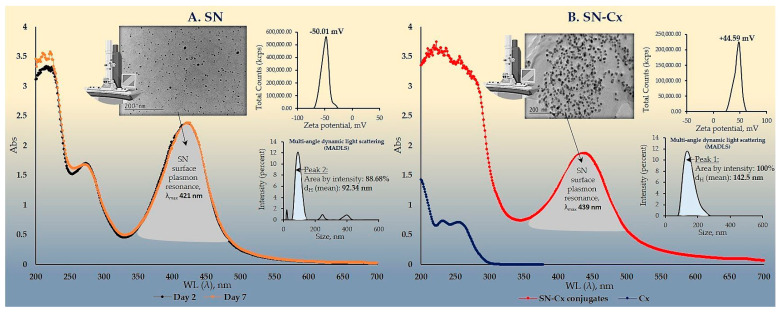
UV-Vis spectra, TEM micrographs, zeta potential distribution, and size analysis via MADLS of: (**A**) SN sample 2C8R4.5; (**B**) SN-Cx sample 2C8R4.5Cx+++.

The electron microscopy and the dynamic light scattering results confirmed a significant enlargement of the conjugated particles’ diameter and hydrodynamic diameter (d_H_)m compared to the SN alone. These observations are in accordance with the shift in the SPR peak of the conjugates to higher values (SN λ_max_ = 421 nm < SN-Cx λ_max_ = 439 nm). Macroscopically, the conjugated nanosuspension generally retained the visual appearance of the unconjugated sample but presented slightly more pronounced opalescence. Negligible amounts of sediment were repeatedly observed after a few days of storage but the sediment was easily resuspended upon shaking. Figure 2A,B shows the UV spectra, TEM micrographs, zeta potential distribution, and size analysis by the hydrodynamic diameter of SN sample 2C8R4.5, and the optimal SN-Cx conjugated form, namely, 2C8R4.5Cx+++.

### 3.2. FTIR Spectroscopy

**Figure 3:**
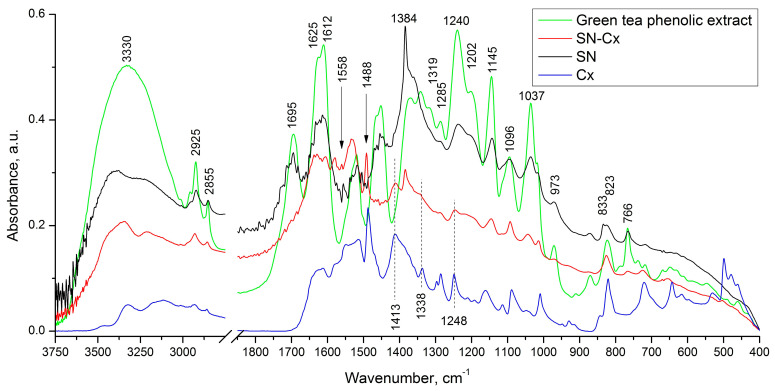
FTIR spectra of green tea phenolic extract (green line), SN (black line), Cx (blue line), and SN-Cx (red line) samples.

In the infrared spectrum of the phenolic extract (Figure 3), multiple bands of relatively high intensity, which are characteristic of the four catechin diastereoisomers, were observed at 1627, 1625, 1612, 1520, 1240, 1202, 1145, 1096, and 1037 cm^−1^ [39]. Bands falling in the range of 1400 to 1650 cm^−1^ are considered to be characteristic of the catechin’s aromatic (benzenoid) residues [40]. In the same spectrum, the remaining high-intensity bands (with maxima at 1037, 1145, 1240, and 1285 cm^−1^) are associated with the inherent phenolic and alcohol groups of C–O stretching and δ(O–H) vibrations, and those registered at the higher wavelengths (at 766 and 823 cm^−1^) are associated with the out-of-plane C_ar_–H modes.

In the spectrum of the SN sample, the presence was observed of all the absorption bands mentioned above, which are characteristic of the phenolic extract used. Regarding the reason for the preserved IR catechin imprint in the spectrum of the SN sample, we would suggest the tendency of catechins to sorb on the surface of metal nanoparticles that were formed with their participation [29]. It should be noted that in the spectrum of the sample in question, the appearance of bands characteristic of the asymmetric and symmetric stretching vibrations of NO_2_ groups (at 1558 and 1384 cm^−1^) was also registered. As an additional but debatable indication of the presence of the latter, we would point to the presence of the low-intensity band at 833 cm^−1^, which represents a band characteristic of C_ar_–NO_2_ oscillations. The reason for the existence of this functional group will not be discussed here, but it is assumed that in the course of the imposed redox process, the nitration of the organic substrate also takes place.

Moreover, as shown when comparing the spectra of the Cx and SN-Cx samples, the presence of the used anti-infective drug in the composition of the target nanoparticles was established.

### 3.3. X-ray Diffraction (XRD)

**Figure 4:**
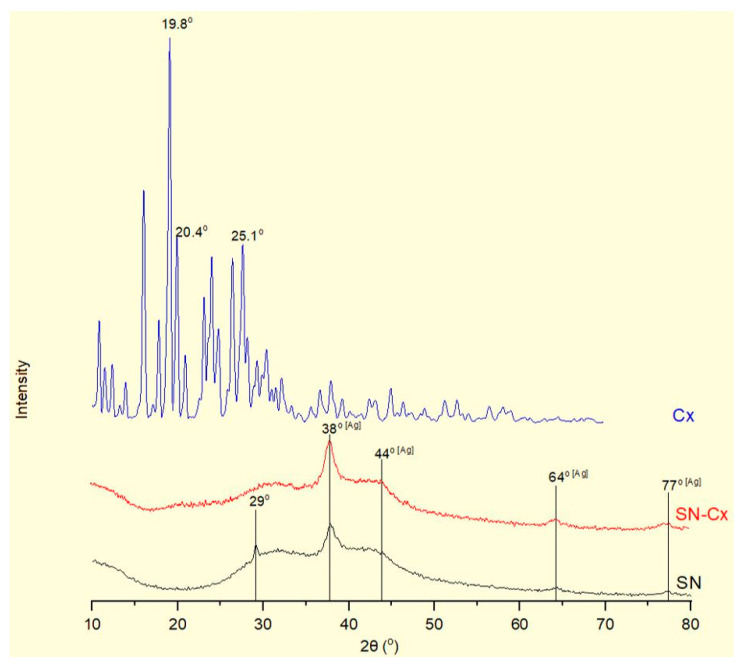
XRD of the SN sample 2C8R4.5 (black line), SN-Cx sample 2C8R4.5Cx+++ (red line), and chlorhexidine diacetate (blue line).

The X-ray diffractograms of the silver nanoparticles (SN), their conjugates with chlorhexidine (SN-Cx), and chlorhexidine acetate (Cx) are shown in Figure 4. Recognizable in the conjugated and non-conjugated nanoparticles’ spectra is the standard diffraction pattern of elemental silver (Ag), with face-centered cubic symmetry. The latter is distinguished by reflections at 2θ 38°, 44°, 64°, and 77°, corresponding to the (111), (200), (220), and (311) planes [41,42,43,44]. The broad scattering and the lack of sharp, intense peaks in the SN and SN-Cx spectra reveal the substantial presence of amorphous matter and the small crystallite size [41,43]. None of the intrinsic chlorhexidine diffraction peaks (e.g., 2θ 19.8°, 20.4°, and 25.1°) [45,46,47] was evident in the conjugated nano-silver form; this observation confirms the amorphization of the drug upon loading onto the metal delivery platforms. The presence of crystalline silver oxide—Ag_2_O, the recognized standard being intense reflections at 28° and 32° of the (110) and (111) planes [48,49,50]—was not detected in the nano samples. However, an extra peak was recorded in the X-ray spectrum of SN at 29°, which might be a result of catechin crystalline segments in the sample [51], which are spectrally disguised or amorphized in the presence of chlorhexidine.

### 3.4. Antibacterial and Antifungal Activity

#### 3.4.1. Minimal Inhibitory Concentration (MIC)

The inhibitory activity of SN alone against *E. coli* was recorded at relatively high concentrations of 175 µg/mL (e.g., dilution 1:4); antimicrobial activity against the other two pathogens—*S. aureus* and *C. albicans*—did not manifest at the investigated concentrations. It is worth mentioning that a slight opalescence was observed even within the negative controls with SN, which directed our attention to the MBC/MFC results. Such an opalescence is likely a result of the colloidal instability of the SN samples in the electrolyte-rich fluid growth medium.

When Cx was tested alone, MICs of 35.3 µg/mL (dilution 1:32) against *E. coli* and 17.7 µg/mL (dilution 1:64) against *S. aureus* and *C. albicans* were established. The SN-Cx conjugates exceeded these results by demonstrating MIC against all strains at SN 5.5 µg/mL + Cx 8.8 µg/mL (dilution 1:128). Only vials from the last in-row tested concentrations, corresponding to the 1:256 dilutions, remained turbid. The negative controls here were clear and transparent.

#### 3.4.2. Minimal Bactericidal/Fungicidal Concentration (MBC/MFC)

**Figure 5:**
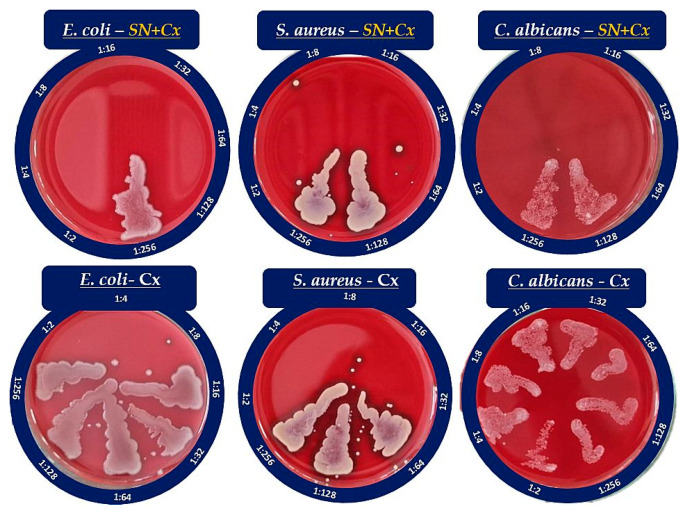
Seeds of the Cx and SN-Cx diluted suspensions on blood agar.

The MBC/MFC study confirmed the lack of antimicrobial activity of SN alone against *S. aureus* and *C. albicans*, whereas the established MIC against *E. coli*, 175 µg/mL, was also shown to correspond to the MBC. The results from the MBC/MFC tests of the Cx and SN-Cx conjugates are presented in Figure 5 and are summarized in Table 3.

According to these results, the conjugates (SN-Cx) demonstrate synergism against *E. coli*, *S. aureus*, and *C. albicans*. Due to the lack of established activity of SN alone against *S. aureus* and *C. albicans*, the interaction therein could be further defined as potentiation [52]. Additionally, we have confirmed the observations of other authors that Gram-negative microorganisms are, in general, more susceptible to the presence of silver nanoparticles than Gram-positive bacteria, which is likely due to the thicker cellular wall–peptidoglycan layer of the latter and the consequently restricted penetration of Ag^+^ [53,54].

### 3.5. Cytotoxicity, Antiviral, and Virucidal Activity

The results for CC_50_, IC_50_, SI, and virucidal activity (Δlg) of the SN, Cx, and SN-Cx samples are presented in Table 4, Table 5 and Table 6.

The as-determined CC_50_ values of SN in MDCK, A549, and BJ cell cultures—56.4, 60.7, and 16.9 μg/mL, respectively—reveal SN as an active agent with relatively low to moderate toxicity. In MDCK cells, the CC_50_ of Cx alone was 2.6 and was 2.5 μg/mL in the conjugated form of SN-Cx. In A549 cell cultures, the same respective values were 5.3 and 5.8 μg/mL, while in human BJ fibroblasts, they were 2.2 and 2.4 μg/mL. According to these results, Cx and the Cx-bearing conjugated form (SN-Cx) could be defined as agents with high toxicity. SN showed a weak effect on the replication of the A/Aichi/2/68 virus, with a mean IC_50_ = 43.6 μg/mL and SI = 1.29. In the applied concentrations, the Cx and SN-Cx samples did not affect the replication of the Influenza virus A/Aichi/2/68 (H3N2) in MDCK cell cultures in a dose-response experimental design.

A strong virucidal effect was established at an SN MTC of 35 μg/mL with a duration from 5 to 60 min. The absence of a virucidal effect of Cx and SN-Cx against the investigated Influenza strain A/Aichi/2/68 (H3N2) is probably due to the low working concentrations of 2 μg/mL for Cx and 1.2/2 μg/mL for SN-Cx, respectively, which were applied because of the substrates’ high toxicity.

The distinguished time-dependent virucidal effect of SN was also confirmed against the Influenza strain A/Panama/2007/99 (H3N2), at a working concentration of 350 μg/mL upon contact and following ten-fold dilution prior to titration in the cell monolayer. The virucidal activity of Cx and SN-Cx against the Influenza A/Panama/2007/99 (H3N2) virus was assessed as weak (5 and 15 min) to moderate (30 and 60 min) when higher concentrations (than those in the former experiment with the A/Aichi/2/68 (H3N2) strain) were in contact with the virus, namely, Cx 113.0 μg/mL and SN-Cx 70.0/113.0 μg/mL, respectively. Before titration in the cell monolayer, additional ten-fold dilutions were still performed so that non-toxic concentrations were applied. However, the residual toxicity of the Cx and SN-Cx preparations in the greater dilutions and respective interference with the CPE could explain the established lower virucidal activity compared to that in SN alone.

## 4. Conclusions

Chlorhexidine diacetate was successfully adsorbed onto the “green”-synthesized silver nanoparticles’ negatively charged surface. The thus-obtained colloidal solution retained satisfactory physical stability for the duration of the survey. The resultant conjugates presented enhanced antimicrobial activity, judging by the results for common pathogens such as *Escherichia coli*, *Staphylococcus aureus*, and *Candida albicans.* The addition of nano-silver to chlorhexidine solution significantly decreased the minimal bactericidal/fungicidal concentrations for all tested bacteria and fungi. Moreover, the fungicidal activity of chlorhexidine alone against *C. albicans* was not established at the investigated concentrations, whereas the unconjugated silver nanoparticles were not shown to inhibit or kill *S. aureus* and *C. albicans* independently. As expected, the cationic surfactant chlorhexidine exhibited high toxicity on the Madine–Darby canine kidney line, human fibroblasts, and lung carcinoma cells; this was despite the much lower applied active concentrations (0.00004–0.11%) compared to those in commercial topical antiseptic products (0.02–0.2%). The effect did not attenuate upon the conjugation of the drug to nano-silver vehicles, which limited the active concentration range to be tested for antiviral and virucidal activity on Influenza type A. The unconjugated silver nanoparticles, on the other hand, demonstrated good tolerability and low toxicity. Therefore, a relatively high concentration of 35 μg/mL was applied and showed a weak antiviral effect but distinct virucidal properties. Regardless of the latter ascertainments, the chlorhexidine–silver nanoparticle conjugate exhibited a pronounced synergistic action and an extended spectrum of antimicrobial activity. Therefore, it is relevant to test the applicability and efficacy of this promising newly synthesized agent in the form of pharmaceutical dosage for dermal, nasal, oromucosal, and ocular administration. This prospect will require more specific and bio-relevant tolerability tests to be conducted on 3D and organoid cultures.

## Figures and Tables

**Table 1:** Silver nanoparticle test formulations and UV-Vis spectral results.

Formulation Code	Silver Nitrate Conc., mM	Reductant (C8) conc., mg/mL *	Day 2		Day 7	
			λmax, nm	Abs ± SD	λmax, nm	Abs ± SD
1C8R0.6	1	0.6	426	0.307 ± 0.001	430	0.481 ± 0.001
2C8R0.6	2	0.6	425	0.435 ± 0.002	429	0.571 ± 0.001
1C8R1	1	1.0	423	0.617 ± 0.001	429	0.744 ± 0.002
2C8R1	2	1.0	425	0.776 ± 0.002	428	0.889 ± 0.002
1C8R1.5	1	1.5	425	0.753 ± 0.002	429	0.913 ± 0.002
2C8R1.5	2	1.5	425	0.948 ± 0.001	430	1.125 ± 0.003
1C8R3	1	3	417	1.350 ± 0.003	418	1.395 ± 0.005
2C8R3	2	3	421	1.828 ± 0.003	424	1.904 ± 0.002
1C8R4.5	1	4.5	416	1.379 ± 0.004	415	1.367 ± 0.003
**2C8R4.5**	**2**	**4.5**	**421**	2.374 ± 0.003	**422**	2.386 ± 0.002
1C8R6	1	6	413	1.448 ± 0.004	415	1.498 ± 0.002
2C8R6 **	2	6	417	2.584 ± 0.004	420	2.758 ± 0.003

* The reductant volume was kept constant at 0.5 mL. ** Turbidity was recorded and a slight sediment was formed after a few days of storage.

**Table 2:** Zeta potential of the SN and SN-Cx samples.

Formulation Code	Cx 3.75 mg/mL vol. (mL)	Cx Final Conc. in Solution, mg/mL	Zeta Potential, mV
2C8R1.5	n/a *	n/a	−43.45
2C8R3	n/a	n/a	−39.06
**2C8R4.5**	**n/a**	**n/a**	**−50.01**
2C8R1.5 Cx+	0.5	0.47	+31.24
2C8R1.5 Cx++	1.0	0.83	+33.74
2C8R1.5 Cx+++	1.5	1.13	+36.2
2C8R3 Cx+	0.5	0.47	+40.02
2C8R3 Cx++	1.0	0.83	+44.34
2C8R3 Cx+++	1.5	1.13	+42.44
2C8R4.5 Cx+	0.5	0.47	+41.22
2C8R4.5 Cx++	1.0	0.83	+43.78
**2C8R4.5 Cx+++**	**1.5**	**1.13**	**+44.59**

* n/a—not applicable.

**Table 3:** MICs and MBC/MFCs of SN, Cx, and SN-Cx, along with the FICs of SN and Cx.

Infectious Strain	MIC			MBC/MFC			FIC_SN_	FIC_Cx_
	SN (µg/mL)	Cx (µg/mL)	SN + Cx (µg/mL)	SN (µg/mL)	Cx (µg/mL)	SN + Cx (µg/mL)		
*E. coli*	175	35.3	SN 5.5 + Cx 8.8	175	141.3	SN 5.5 + Cx 8.8	0.031	0.023
*S. aureus*	n.e.*	17.7	SN 5.5 + Cx 8.8	n.e.	35.3	SN 10.9 + Cx 17.7	<<0.016 **	0.497
*C. albicans*	n.e.*	17.7	SN 5.5 + Cx 8.8	n.e.	n.e.	SN 10.9 + Cx 17.7	<<0.016	0.497

* n.e.—not established. ** MICs of SN against *S. aureus* and *C. albicans* were not recorded in the investigated concentration range; thus, when this MIC value was needed for the FIC formula, it was considered to be higher than the maximal applied concentration, i.e., >350 µg/mL.

**Table 4:** Cytotoxicity in MDCK, A549, and BJ cells and antiviral activity in an experimental infection with 100 CCID_50_/mL of *Influenza* virus A/Aichi/2/68 (H3N2) from the MDCK line.

Active Agent	Cell Line	Cytotoxicity	Antiviral Effect	
		CC_50_ (μg/mL) ± SD	IC_50_ (μg/mL)	SI
SN	MDCK	56.4 ± 0.4	43.6 ± 16.6	1.29
Cx	MDCK	2.5 ± 0.7	-	-
SN-Cx	MDCK	1.6/2.6 ± 0.1	-	-
SN	A549	60.7 ± 0.3	n.d.	n.d.
Cx	A549	5.3 ± 0.7	n.d.	n.d.
SN-Cx	A549	3.6/5.8 ± 0.2	n.d.	n.d.
SN	BJ	16.9 ± 0.4	n.d.	n.d.
Cx	BJ	2.2 ± 0.1	n.d.	n.d.
SN-Cx	BJ	1.5/2.4 ± 0.5	n.d.	n.d.

SD—standard deviation. n.d.—not determined.

**Table 5:** Virucidal activity at the maximal tolerated concentrations (MTC) of SN, Cx, and SN-Cx against 100 CCID_50_/mL of *Influenza* virus A/Aichi/2/68 (H3N2) and titration from the contact sample in MDCK cells.

Active Agent	Δlg			
	5 min	15 min	30 min	60 min
SN 35 μg/mL	2.5	2.66	3.00	3.00
Cx 2.0 μg/mL	0	0	0	0
SN-Cx 1.2/2.0 μg/mL	0	0	0.33	0.33

**Table 6:** Virucidal activity of SN 350 μg/mL, Cx 113 μg/mL, and SN-Cx 70.0/113.0 μg/mL against 100 CCID_50_/mL of *Influenza* virus A/Panama/2007/99 (H3N2) and titration from a 10-fold dilution of the contact sample in MDCK cells.

Active Agent	Δlg			
	5 min	15 min	30 min	60 min
SN 350 μg/mL	2	2.33	2.5	3
Cx 113 μg/mL	1	1.33	1.66	1.66
SN-Cx 70.0/113.0 μg/mL	1	1.33	1.66	2

## Data Availability

Not applicable.
